# Photonic Bound States in the Continuum in Nanostructures

**DOI:** 10.3390/ma16227112

**Published:** 2023-11-10

**Authors:** Hongkun Zhong, Tiantian He, Yuan Meng, Qirong Xiao

**Affiliations:** State Key Laboratory of Precision Measurement Technology and Instruments, Department of Precision Instrument, Tsinghua University, Beijing 100084, China; zhk23@mails.tsinghua.edu.cn (H.Z.); htt21@mails.tsinghua.edu.cn (T.H.); mengy16@tsinghua.org.cn (Y.M.)

**Keywords:** bound states in the continuum, nanostructures, 2D materials

## Abstract

Bound states in the continuum (BIC) have garnered considerable attention recently for their unique capacity to confine electromagnetic waves within an open or non-Hermitian system. Utilizing a variety of light confinement mechanisms, nanostructures can achieve ultra-high quality factors and intense field localization with BIC, offering advantages such as long-living resonance modes, adaptable light control, and enhanced light-matter interactions, paving the way for innovative developments in photonics. This review outlines novel functionality and performance enhancements by synergizing optical BIC with diverse nanostructures, delivering an in-depth analysis of BIC designs in gratings, photonic crystals, waveguides, and metasurfaces. Additionally, we showcase the latest advancements of BIC in 2D material platforms and suggest potential trajectories for future research.

## 1. Introduction

Bound states in the continuum (BIC) in photonic structures have emerged as a pivotal concept with vast applications in optics and photonics [1,2,3]. By judiciously designing the potential structure, BIC was first constructed mathematically in 1929 by von Neumann and Wigner as resonant states existing within the radiation continuum that does not radiate [4]. Since the BIC concept was further refined by Stillinger and Herrick [5], the wisdom has been widely accepted and applied to various wave phenomena, such as acoustic waves [6,7,8,9], water waves [10,11,12], and electromagnetic waves [13,14,15]. After the first observation of BIC in optical systems [13,16], this nascent topic has witnessed rapid development and expansion in photonics [17,18,19,20].

**Figure 1:**
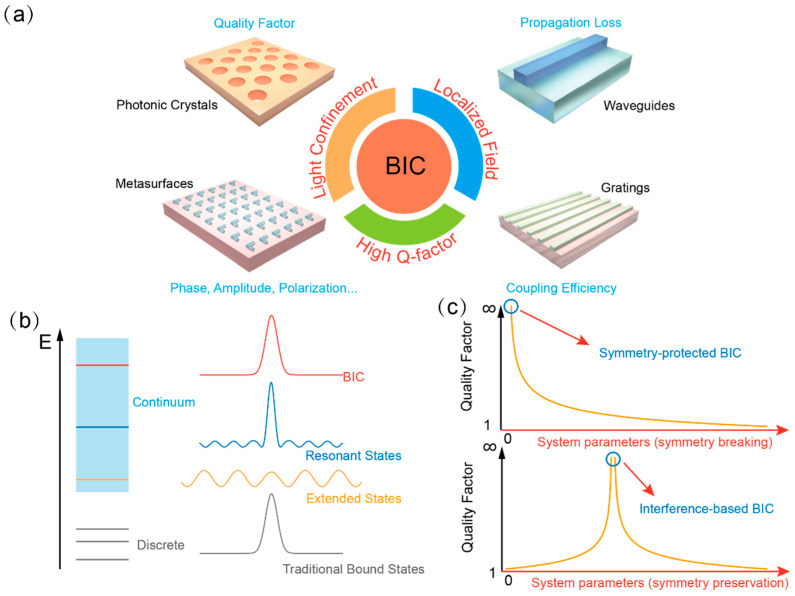
(**a**) A graphical depiction highlighting the core characteristics of BIC (annotated in red) and the associated enhancement in various photonic structures (annotated in blue). (**b**) A concise depiction of the BIC concept, inspired by Ref. [2]. (**c**) A plot comparing the quality factor against system parameters, illustrating the differences between symmetry-protected and interference-based accidental BIC. Adapted with permission from Ref. [42].

The high-quality factor, strongly localized field, non-radiative property, unique light confinement mechanism, and intriguing topological nature of BIC have infused new vigor into areas such as resonator design [21,22,23,24], low-loss optical transmission [18], efficient nonlinear generation [25,26], and advanced light–matter interactions [27,28,29] by applying BIC within diverse nanostructures [30,31,32,33]. Figure 1a gives an illustrative representation of the core features of BIC highlighted in its surrounding circle. The adjacent photonic structures depict the enhanced properties derived from the incorporation of BIC. In gratings, BIC can significantly enhance light–matter interactions [34], leading to more efficient diffraction and light manipulation [35]. Photonic crystals with periodic structures can leverage BIC to achieve complete bandgap properties, further fine-tuning light propagation and confinement [36]. Waveguides also benefit from BIC to design long-lived resonances, optimizing light transmission with minimal loss [18]. Meanwhile, metasurfaces, known for their ability to manipulate electromagnetic waves on subwavelength scales, can pair with BIC to realize sharp resonances [37] and improved control over wavefronts [38]. By harnessing BIC’s superior attributes across these nanostructures, researchers have unlocked new horizons in photonic applications and innovations [1,39]. Furthermore, the synergy of BIC with 2D materials also enhances the performance of versatile optoelectronic devices such as dynamic switching ability [40], harmonic generation efficiency [34], and photoluminescence (PL) intensity [41] and provides opportunities for observing novel physical phenomena like polariton-induced nonlinearity [26] and collective behavior of Bose–Eistein condensate [32].

In this review, we focus on recent advancements in integrating BIC concepts within nanophotonics, organizing them by distinct photonic structures to highlight the boosted functionalities infused with BIC. The fundamental physics of BIC and prevalent analytical tools are also discussed.

## 2. Fundamentals of BIC

### 2.1. Classification of BIC

A burgeoning body of literature has emerged in recent years to discuss the fundamentals of BIC [1,3,39], reflecting the profound interest in this intriguing optical phenomenon. As demonstrated in Figure 1b, photonic BIC refers to an electromagnetic mode that is confined to a finite region of space without radiating, similar to a traditional bound state, but exists within the continuum spectrum of energy or frequencies that only permits extended states with inevitable radiation loss in the traditional situation [4,5]. We here focus primarily on the two most prevalent scenarios: symmetry-protected BIC and interference-based BIC, because the two categories represent the cornerstone of current BIC research. A comprehensive understanding of their characteristics and underlying mechanisms is crucial. For other BIC types like separability-induced BIC and inverse-construction-based BIC, introductions can be found in previous studies [2,5,43,44].

**Figure 2:**
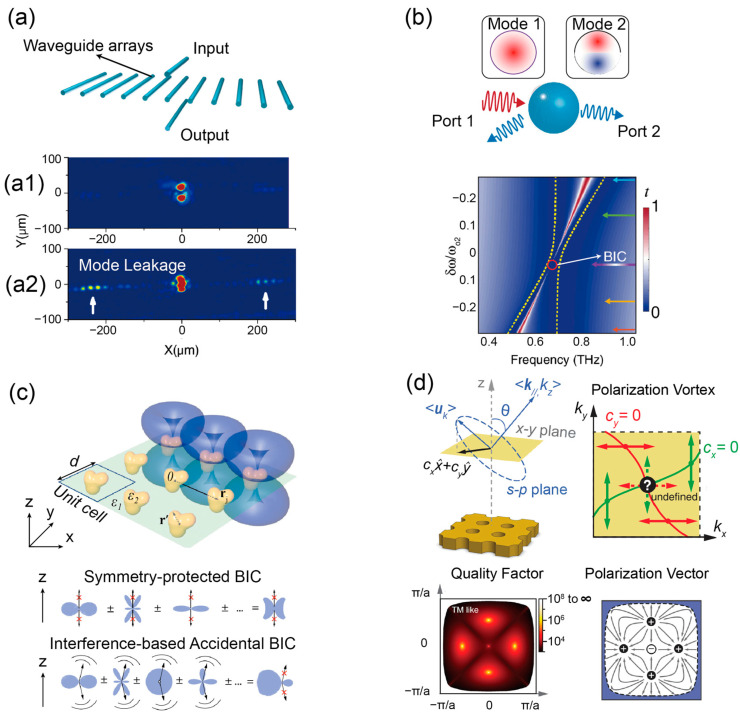
Insights into BIC mechanisms and analytical techniques. (**a**) Symmetry-protected BIC in waveguide arrays. (**a1**): Horizontally aligned waveguide arrays with two supplementary vertical waveguides. (**a2**): Visual distinction between the localized field in symmetry-protected BIC and the mode leakage upon y-symmetry disruption. Adapted with permission from Ref. [14]. (**b**) Illustration of interference-based BIC. Upper: Depiction of interference-based BIC highlighted by two radiative modes. Lower: Resonance transmission variations based on frequency shifts, marking BIC at a peak reflectance point with red circles. Adapted with permission from Ref. [71]. (**c**) Multipole expansion in square lattice metasurfaces. Lower: Dual visuals explaining BIC formations using multipole expansion. Adapted with permission from Ref. [78]. (**d**) Topological characteristics of BIC in photonic crystals. Upper: Breakdown of radiation fields in PhC slabs and the associated nodal lines for polarization vector components in k-space near BIC. Lower: Calculations of Q-factor and polarization direction within the k-space. Adapted with permission from Ref. [79].

*Symmetry-protected BIC*. A state qualifies as a symmetry-protected BIC when it cannot couple to leakage modes due to a symmetry mismatch. Consequently, this state manifests as a bound state, not radiating even within the continuum band. Figure 2a provides an illustrative example of this phenomenon [14]. The systems consist of horizontally aligned waveguide arrays with two supplementary vertical waveguides (Figure 2a). With careful design, the horizontally arranged array was optimized to support a guide band with modes that exhibit symmetry in the y-direction. Conversely, the two vertically aligned arrays were tailored to support modes with y-directional anti-symmetry, and their frequencies are precisely located within the guide band. Within the guide band, modes from the vertically arranged array should ideally transmit within the horizontal array. However, in practice, a highly localized electromagnetic field was observed in the vertically aligned arrays, as depicted in Figure 2(a1). Additionally, upon introducing a ΔT = 30 K temperature gradient into the vertical waveguides to disrupt the y-anti-symmetry, it was observed that the mode within the vertically arranged array resumed its transmission, as depicted in Figure 2(a2). Such contrast phenomenon can be explained by the BIC concept in Figure 1a. In the prior scenario, energy transmission was suppressed due to a y-symmetry mismatch, leading to a localized bound state even within the continuum guide band. However, once the y-anti-symmetry of the modes in the vertical arrays is disrupted, they are no longer protected from the symmetry mismatch, causing them to degrade into resonant states with mode leakage in horizontal arrays. Currently, the development of symmetry-protected BIC mainly relies on electromagnetic field modes with specific symmetries in certain periodic structures such as gratings [45], photonic crystals [46,47,48], and metasurfaces [49,50,51]. For instance, the well-known Γ BIC in photonic crystal with C_4v_ symmetry can be attributed to C_2_ symmetry mismatch between odd and even modes at frequencies below the first-order diffraction limit [52] (*ω* < 2π/d).

Analogous to the introduction of a temperature gradient in Figure 2(a2), the deliberate incorporation of defects to disrupt the inherent symmetry causes a perfect symmetry-protected BIC to degenerate into a quasi-BIC that interacts with continuum modes [53]. This interference between a discrete localized quasi-BIC and a continuum band of states results in a characteristic Fano resonance lineshape in the transmission spectrum [54] with a considerable high-quality factor [55,56,57,58]. Due to the high-quality factors, the transmission spectrum presents a sharply defined Fano resonance peak, which has been instrumental in the development of various sensors [59,60,61,62]. In addition, symmetry-protected BIC is resilient to perturbations that maintain the underlying symmetry, hence exhibiting a greater tolerance for manufacturing errors; therefore, they have been widely applied in ultra-high speed light manipulation [30], imaging [63,64,65], and low-threshold lasers [21,66].

*Interference-based BIC.* An interference-based BIC arises due to the destructive interference of individual radiation channels in resonant structures by judiciously tunning parameters. A unique characteristic of interference-based BIC is its emergence when the electromagnetic modes lack symmetry [17], as opposed to the previous situation. To achieve interference cancellation, the number of tunning parameters typically needs to surpass the radiation channels. However, as the parameter count rises, tuning becomes increasingly complex, making this method more effective with fewer radiation channels [67,68,69,70]. Figure 2b illustrates an example involving two radiation channels [71]. Here, one mode becomes more lossy, while the other displays a total reflection behavior in the spectrum, characteristic of a BIC state.

According to the spatial relationship among resonant cavities, interference-based BIC can be subdivided into Fabry–Perot (F-P) type BIC and Fredrich–Wintgen type BIC [2]. F-P BIC originates from destructive interference between two resonant cavities spatially separated by a specific interval. Given that resonance can be viewed as a perfectly reflective mirror at its resonant frequency when linked to a single radiation channel, an F-P BIC can be established by adjusting the distance between two such resonant structures to accumulate a phase shift equivalent to a whole number multiple of 2π. This BIC is named for its similarity to the F-P resonator design methodologies and has been observed in various photonic systems [72,73].

Notably, a specific distance between the two resonances is not a strict requirement. When two resonances occupy the same spatial location, a BIC can still be realized if they are accurately coupled via the same radiation tunnel, as demonstrated by Fredrich and Wintgen in 1985 [74] using the temporal coupled-mode theory [2,75,76]. This is referred to as the Fredrich–Wintgen type BIC. This variant of BIC is distinct from the former one due to the near-total reflection observed close to the intersection of two uncoupled spectral lines [69,71,77].

### 2.2. Multipole Expansion

The multipole expansion method offers a systematic approach to represent intricate wave fields by decomposing them into localized source terms, including monopoles, dipoles, and higher-order multipoles [80]. This decomposition not only simplifies complex fields but also provides a comprehensive insight into wave phenomena. Historically, the method has been instrumental in areas such as the Mie scattering theory [81,82,83], antenna theory [84,85], and resonance design [86,87].

Analyzing the multipole components of BIC states offers a fresh perspective on the origins of BIC [78]. The rise of symmetry-protected BIC is linked to the non-transverse radiation characteristics of the multipole components, and the accidental off-Γ BIC stems from the destructive interference effect between various multipole orders, as shown in Figure 2c. Valued for its capacity to highlight dominant multipole modes, the multipole expansion method has been extensively used in BIC design for metasurfaces and photonic crystals [37,57,88], which aids in comprehending the far-field radiation behavior of BIC modes [22,89].

### 2.3. Topological Origin

Inspired by condensed matter physics, topological photonics aims to design and harness optical structures that possess unique properties derived from their topological nature. This field has witnessed significant advancements in recent years [90,91,92]. Due to their robustness against fabrication defects and noise, a range of photonic devices leveraging topological principles has been developed, including topological microcavity polariton lasers [93,94] and signal transporters facilitated by the terahertz quantum valley Hall effect [95]. Additionally, the topological method offers a fresh lens to comprehend the BIC mechanism. Pioneering works have revealed that BIC in photonic crystals aligns with vortex centers, carrying quantized topological charges in the polarization far-field space [79] (Figure 2d). Subsequent research has expanded these findings to periodic arrays of dielectric spheres [96] and one-dimensional (1D) gratings system [89].

The inherent topological properties of BIC in momentum space present a unique approach to generating high-purity, efficient vortex beams by leveraging its quantized topological charges and suppressed-side-radiation characteristics [22,97,98,99,100]. Furthermore, dynamic switching capabilities have been achieved using a subwavelength-thin phase-change halide perovskite BIC metasurface, which allows for the alteration of emission patterns between polarization vortices with opposing topological charges at distinct wavelengths [40,101].

## 3. BIC in Various Photonic Structures

### 3.1. Gratings

Optical gratings are periodic structures that can manipulate the direction and wavelengths of incident light waves in specific ways. With their ability to disperse incident light, optical gratings provide a flexible platform for engineering BIC [102,103]. 

**Figure 3:**
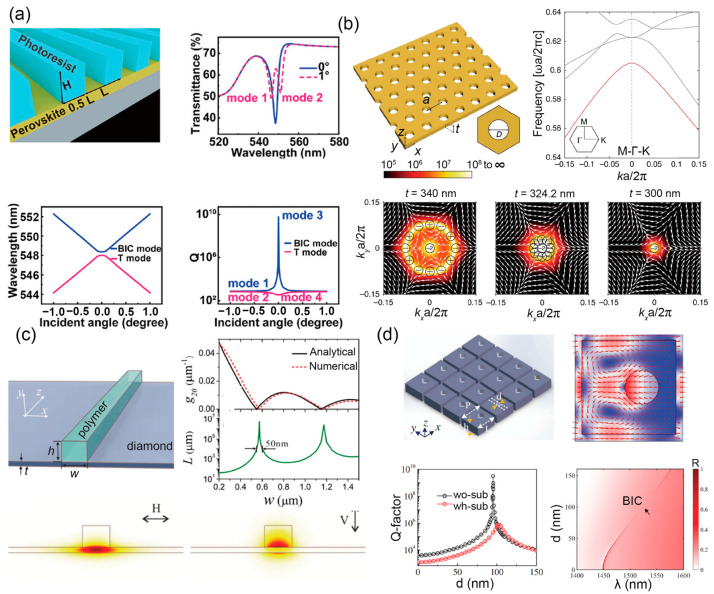
Exploration of BIC in diverse nanostructures. (**a**) BIC-based grating perovskite microlaser. Upper: Representation of the grating structure alongside a transmission spectrum for two incident angles. Lower column: Correlation between mode wavelength and Q-factor based on incident angles for multiple modes. Adapted with permission from Ref. [112]. (**b**) Photonic crystal slab BIC—analyzing changes in k-space with varied slab thickness. Adapted with permission from Ref. [148]. (**c**) BIC-assisted light propagation in waveguides. Upper: Representation of the waveguide structure and graphs showcasing the relationship between coupled strength, propagation distance, and waveguide width. Lower: Depiction of the waveguide’s cross-sectional mode profile. Adapted with permission from Ref. [18]. (**d**) Metasurface BIC highlighting field distribution within the unit cell. Lower: Analysis of the quality factor versus offset distance for a suspended (black) case, a glass substrate (red) case, and reflectance fluctuations in proximity to BIC in a suspended case. Adapted with permission from Ref. [37].

With carefully designed groove spacing [45,104,105], geometry [19,106] and dielectric constant [107,108], one can easily tailor the resonance conditions of BIC to achieve customized control over specific wavelengths or frequency ranges, which bears immense potential in applications such as various environmental sensors [45,60,109], narrowband filters [35,110], and microlasers [111,112] (Figure 3a). Stemming from the destructive interference between two modes, the perovskite microlaser in Figure 3a sustains an interference-based BIC at a normal incident angle with a quality factor approaching 10^10^ and an observed lasing action in 548.5 nm when the pump density reaches 49 μJ/cm^2^ [104]. Beyond its considerable quality factor, the union of BIC with periodic grating also leads to the high confinement of electromagnetic fields within a specific localized area. This enhanced field confinement leads to stronger light–matter interactions [113] exhibiting a typical Fano resonant line shape [45] and also dramatically enhances optical harmonic generation with a boost of several orders of magnitude [34,114].

Beyond simple gratings, there have emerged compound structures broadening the boundaries of applications. Take asymmetry dual-gratings as an example: this kind of grating consists of two parallel gratings with different adjacent gaps separated by a fixed distance, adding new design dimensions to be exploited for controlling quality factor and operating wavelength [115,116,117,118]. The composite integration of waveguides and gratings is another common approach to harness the unique characters of both platforms. The incident light can couple with guide mode in waveguide [119,120,121], owning to the tangential wave vector provided by discrete periodic gratings [122,123,124], namely, the Guide Mode Resonance (GMR) phenomenon. By synergizing the GMR condition with the BIC concept, the Goos-Hänchen shift [125,126] and spin Hall effect [127] can be greatly enhanced due to an ultra-high-quality factor provided by BIC modes. Gratings are also compatible with two-dimensional (2D) materials [128,129,130] such as graphene, perovskite, and transition metal dichalcogenides (TMDs) [131,132,133,134,135,136]. Some novel devices based on these materials have been prototyped by harvesting their tunability [137,138], nonlinearity [34], exceptional gain coefficients, and high quantum yields [112].

### 3.2. Photonic Crystals

Photonic crystals (PhCs), also known as photonic bandgap materials, have been a captivating area of research in photonics for decades [139,140]. These nanostructured materials possess unique optical properties that arise from their ability to control the propagation of electromagnetic waves through periodic modulation of the refractive index [141]. While under the extensive exploration of BIC, this exotic optical concept breaks the traditional wisdom that provides an alternative way to confine light and achieve high-quality resonance in PhCs in addition to the photonic bandgap design method [16]. By capitalizing on the unique properties of both BIC and PhCs, a new generation of compact, efficient, and versatile photonic devices has been prototyped ranging from low threshold lasers [21] to second harmonic generation [25], and other intensively active research field [142].

Composed of a series of periodic dielectrics, 1D PhCs are relatively convenient for designing BIC due to their simple structure ready for exploring conditions for supporting BIC in 1D PhC systems [143,144,145]. Applying BIC in 1D PhCs has enabled enhanced light–matter interactions [28], but other application-oriented research remains relatively scarce and limited by their simplistic structure. For 2D PhC slab systems, BIC can be achieved by judiciously tailoring the lattice geometry parameter [146] and slab thickness [147], exhibiting more design flexibility compared with 1D periodic structure to functionalize a broader range of applications without significantly increasing design complexity. For instance, by harvesting the merits of strong optical field localization, BIC within heterostructure cavity PhC slabs offers an avenue for highly efficient nonlinear frequency conversion [34], which proves to be more pragmatic than directly designing a photonic bandgap at the second-harmonic frequency. The side-radiation suppression property of BIC [20] also enabled some high-performance lasers with lower thresholds to work at room temperature [21,22,24]. Laser beam quality can be further improved by leveraging the topological nature of BIC. As vortex centers exist in far-field polarization fields [79], BIC continuously shifts but does not disappear when changing geometrical parameters. Such robustness allows for merging a cluster of BIC singularities into a single point in momentum space, leading to an enhanced quality factor and better lasing directionality [66,148,149], namely, the merging BIC techniques. Figure 3b provides a comprehensive depiction of the BIC merging process. By adjusting the thickness of a photonic crystal slab exhibiting C_4v_ symmetry, BIC points within momentum space progressively gravitate toward the center with a considerably enhanced quality factor, ultimately culminating in a singular BIC point [148]. Furthermore, coherent complete polarization conversion (CCPC)-empowered feasible polarization control can be achieved by exploiting the strong polarization transition near vortex centers [150,151]. Following a similar approach in designing dielectric PhC BIC, the platform also allows for a more flexible choice of materials, such as van der Waals materials [132,152,153,154] and nearly zero index materials [113,155,156], which may bring intriguing new opportunities.

### 3.3. Waveguides

Photonic waveguides, with their capacity to control light at microscopic levels, act as the primary channels in a circuit. They guarantee the precise direction of photons to the appropriate components at the necessary moments [101,157,158]. Incorporating the principles of BIC into photonic waveguide designs has yielded numerous benefits. Notably, utilizing BIC broadens the material choices available. Traditionally, light confinement in waveguides has been largely achieved with total internal reflection. This method often mandates the use of low-refractive index substrates, limiting the choice of materials for waveguide fabrication [159,160,161]. The inherent characteristic of BIC to sustain localized without decay within a radiative spectrum indicates the potential to use higher-refractive index substrates without leakage, thus preventing unwanted transmission loss [18,162,163,164]. By utilizing BIC, an organic polymer waveguide on a diamond substrate was demonstrated with an ultra-low propagation loss (Figure 3c). Using structural parameter optimization, BIC was realized at specific waveguide widths, exhibiting near-zero coupling strength. This indicates that the bound mode was entirely decoupled from the continuum mode in the waveguide, resulting in the calculated ultra-low propagation loss [18]. Beyond diamond substrates, BIC also facilitates reduced losses in organic polymer waveguides positioned on lithium niobate substrates [165,166], which presents a promising avenue for crafting versatile on-chip integrated photonic devices, including photodetectors [167], modulators [168], and other essential components [169]. Building on this foundation, an on-chip four-channel TM mode (de)multiplexer with data transmission at 40 Gps/channel has been demonstrated for high-dimensional communication [170]. The large nonlinear coefficients and wide transparency window of LiNbO_3_ also suggest that it is an ideal platform for achieving efficient second-harmonic generation [171,172,173]. Some studies have been initiated to explore this capability [169,174].

The giant optical overlap with 2D materials provided by BIC also functionalizes a range of novel hybrid photonics devices. By transferring 2D materials like graphene [167,175], WS_2_ [41] to the lithium niobate substrate before patterning the low-refractive-index waveguides [176], photon emitters [177], switchers, photodetectors, etc., that profit from strong light–matter interactions have been demonstrated in recent years [167]. Other exemplary BIC-enabled applications in waveguide include but are not limited to coherent Fano lasers [178], high sensitivity temperature sensors [179], efficient optical hyperparametric oscillation [180], and diffraction-engineering silicon waveguide grating antennas (SWGAs) [181].

### 3.4. Metasurfaces

Consisting of two-dimensional arrays of subwavelength structures, metasurfaces offer unparalleled capabilities to manipulate light properties [101,122,153,182,183,184], including its amplitude, phase, and polarization [185]. These distinctive traits have been harnessed for diverse applications such as imaging [186], optical computing [187], and optical anticounterfeit [188]. The fusion of BIC’s high Q characteristic with the adaptability of metasurface regulation further amplifies their potential [51,189]. Figure 3d illustrates a BIC within an all-dielectric metasurface. By adjusting the placement of the hollow cylinder inside the unit cell, a specific position can be identified where the mode’s quality factor significantly increases. This surge aligns with an anomaly in the reflection spectrum, signifying the manifestation of a BIC. Incorporating BIC with metasurfaces paves the way for fabricating optical devices with enhanced light-matter interactions, miniaturization, broadband operation, tunability, and reconfigurability. This convergence enhances efficiency across numerous domains, including sensing, imaging, and nonlinear optics.

*Sensing.* By harnessing the high-quality factor property of BIC, metasurface-based sensors [190] can attain unparalleled sensitivity and accuracy. The breakthrough in gold split ring metasurfaces [191] with BIC realized by Srivastava et al. has paved the way for its application in sensing. To further enhance sensitivity, Chen et al. [192] utilized the toroidal dipole bound states in the continuum (TD-BIC) and achieved an impressive amplitude sensitivity of 0.32/RIU. Similarly, Cen et al. [193] demonstrated excellent performance in a refractive index sensor with a sensitivity of 465.74 GHz/RIU and a figure of merit of 32,984. In addition to a single sensing function, more flexible modulation and functions can be explored. A dynamically configurable quasi-bound state in the continuum (QBIC) [194] was proposed using metamaterial arrays with planar symmetric resonators modified with any (active) dielectric, realizing rapid switching of a QBIC resonance with 200% transmission intensity modulation as well as BIC-based refractive index sensing. A variety of high-performance THz sensors based on BIC have been developed by giving a low energy, superior penetrability, and capacity to discern the chemical composition of numerous biological macromolecules inherent in Terahertz waves. These sensors have found applications in a wide range of areas such as thick sensing [191,192], environmental monitoring [193,194], bio-chemical detection [195,196,197,198], optofluidic biosensors [199,200,201], and terahertz devices [202].

*Imaging*. Dynamic imaging and image processing have made rapid advancements with the integration of the BIC and metasurface, along with the utilization of the control of different materials. Yesilkoy et al. [65] demonstrated hyperspectral imaging with dielectric metasurfaces using a metal oxide semiconductor (CMOS) for the spectrometer in 2019. For reconfigurability, Ge_2_Sb_2_Te_5_ (GST) film [203] and graphene [189,204] were used to control dynamic imaging by tuning the resonance or voltage. Meanwhile, image differentiation is a fundamental processing approach for recognizing object edges, and it is frequently the initial step in picture analysis. Therefore, a reconfigurable metasurface was demonstrated [205], embedded in polydimethylsiloxane (PDMS) to situ switch bright-field imaging and 2D differentiation without the need for a Fourier transform. Recently, Wang et al. [206] proposed a multi-channel THz system composed of an Al-graphene programmable metasurface. They used a field-programmable gate array (FPGA) to change the graphene state, which realized the dynamic display of characters in many channels and provided a platform for THz multi-band image encryption and transmission.

*Nonlinear optics.* The strong field confinement and enhanced light–matter interactions provided by metasurfaces open up avenues for investigating nonlinear effects at ultra-low light intensities when coupled with BIC. Metasurfaces are capable of hosting a perfect BIC mode in extremely symmetric geometries. However, it is undetectable. When disrupting geometric symmetry, the ideal BIC would be converted into a quasi-BIC mode, which can be detected. In recent years, the plasmonic metasurface has attained remarkable efficiency, yet it suffers from an inherent dissipative loss. Meanwhile, dielectric and semiconductor materials, which provide lower inherent absorption losses, larger damage thresholds, and stronger nonlinear coefficients, have recently come to be utilized as an alternative.

Nonlinear nanostructured surfaces offer new ways to control and manipulate nanoscale frequency conversion processes in nonlinear optics. The third-harmonic generation (THG) [207], composed of symmetry-broken silicon meta-atoms, was first realized in 2019. In the same year, Xu et al. [208] demonstrated the functional application of dynamical nonlinear image tuning using THG. In addition, methods to improve the second harmonic generation with the slotted silicon nanocube array [209] and the transparent thin-film lithium niobate (LN) metasurface [210] have also been verified. Recently, higher order and harmonics up to the 11th order [211] were confirmed using a resonant dielectric metasurface, which takes nonlinear frequency conversion to a new level.

Semiconductor materials are a crucial part of nonlinear effects. In the visible range, continuous wave (CW) SHG [212] was achieved by combining the attractive material properties of gallium phosphide with higher efficiency but also two orders of pump intensities lower. The entangled photons were generated [213] via spontaneous parametric down conversion by high-Q BIC resonances in gallium arsenide (GaAs) quantum optical metasurface, which paves the way for building room-temperature nanoscale sources of complex tunable entangled states for quantum networks.

Beyond dielectric metasurface systems, the integration of 2D materials [214] in hybrid systems presents numerous novel opportunities. For instance, incorporating graphene into these hybrid metasurface systems facilitates straightforward modulation of the Fano resonance peak via controlling the bias voltage [215,216,217]. This electrically regulated tunability paves the way for dynamic metasurface-driven displays [189,218]. Moreover, as a notable 2D material renowned for its intrinsic nonlinearity and potent luminescence, WS2 can either be layered atop a metasurface or crafted into Mie resonator nanodisks for enhanced second harmonic (SH) intensity [219] and highly directed light emission [220].

## 4. Summary and Perspective

We provided a concise overview of recent progress in BICs, categorizing them by their unique photonic structures. By comparing all the applications in various photonic structures, it is evident that the concept of BIC has facilitated notable breakthroughs in areas like light confinement, resonator design, optical sensing, nonlinear generation, and other intensively studying fields. As an emerging field that is rapidly developing, BIC promises to invigorate the following trends in photonics, potentially indicating the direction of the latest research endeavors.

### 4.1. Chiral BIC

As a fundamental geometrical property, chirality refers to objects or systems where they are distinguishable from their mirror image. In the specific domain of photonics, chirality has garnered substantial interest for its capability of modulating the geometrical phase for arbitrary wavefront shaping [221,222], efficiently manipulating the polarization state of light emission [223,224,225] and advancing light spin manipulation [172,226]. The high-quality factor, strong field enhancement, and far-field directionality properties of BIC align well with the requirements of the aforementioned applications. Therefore, chiral BIC has become one of the recent hotspots in BIC research.

**Figure 4:**
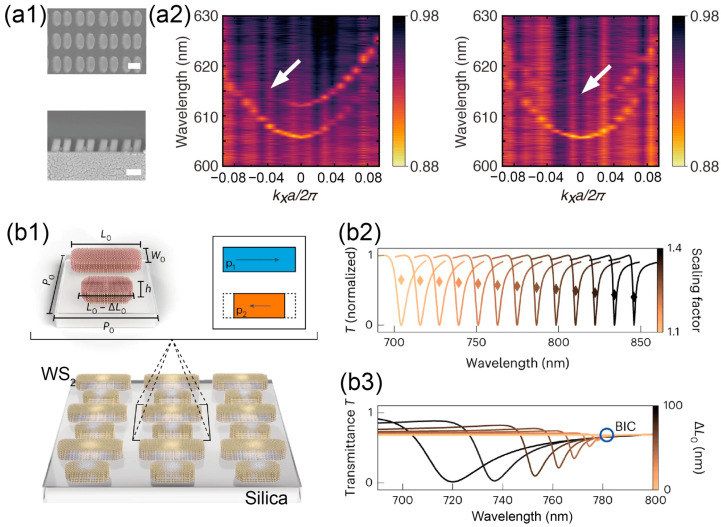
(**a1**) Top-view and side-view SEM for symmetry-broken metasurface. (**a2**) Angle-dependent transmission for LCP (off-Γ, external chirality) and RCP (Γ, intrinsic chirality). (**a1**,**a2**) Adapted with permission from Ref. [31]. (**b1**) Schematic of van der Waals material-based self-hybridization BIC. (**b2**) Spectrum shift by changing scaling factor. (**b3**) Transmission versus wavelength at different defect sizes. BIC is observed when p_1_ = p_2_. (**b1**–**b3**) Adapted with permission from Ref. [27].

Dielectric and plasmonic metasurfaces, with their unique advantages in light manipulation, have emerged as ideal platforms for harnessing chirality [227,228,229]. By breaking in-plane C_2_ symmetry, the well-known Γ symmetry-protected BIC belongs to a square lattice array split into two circular polarization quasi-BIC points that exhibit chirality [48,230,231]. Due to their deviation from Γ points corresponding to normal incident directions, this kind of chiral BIC generally suffers from limited emission performance and a fundamental trade-off between the *Q*-factor and circular dichroism [228]. To address this problem, additional parameters should be introduced either to control the position of the quasi-BIC or to decouple the correlation effect between the quality factor and circular dichroism. By further breaking the out-plane symmetry, the circular quasi-BIC point can be shifted to the Γ point and thus becomes an intrinsic chiral BIC corresponding to normal incidence, offering enhanced performances in chiral emission and lasing [31,38] (Figure 4(a1,a2)). An alternative approach can be considered as exploiting a structure consisting of a 2D twisted vertical split-ring resonator array and a 1D grating. This design can independently generate chirality and maintain a high-quality factor without producing a negative offsetting impact [227].

### 4.2. BIC in Hybrid Structures with 2D Materials

With the advancement in BIC research, the focus has expanded from purely dielectric BIC to include BIC in hybrid systems. This expansion firstly facilitates the intricate interplay between metallic and dielectric materials [232], giving rise to unprecedented optical characteristics. The fusion of plasmons with BIC mainly aims to leverage the high-quality factor property of BIC to address the inherent ohmic losses associated with metal-based systems [233]. By combining metals like gold with dielectrics like SiO_2_ [234], plasmonic BIC can be achieved in such a hybrid system with more compressed modal volume [235] and stronger light–matter interactions [236,237], which has been exploited for biomolecular sensing [236,238].

Another particularly promising avenue is the integration of BIC with 2D materials. The novel functionalities offered by 2D materials were discussed in the prior sections; here, we focus more on enhanced coupling between excitons and optical modes in 2D material hybrid systems. Characterized by large oscillator strengths in their excitons, monolayer TMDs present promising platforms for investigating strong light–matter interactions. By transferring monolayer TMDs onto dielectric metasurfaces [239] or photonic crystal slabs [240], Rabi-splitting can be significantly enhanced due to the intense coupling between the excitons in 2D materials and the optical quasi-BIC in dielectrics. This coupling can be further optimized by adjusting the position of the monolayer and the thickness of the dielectrics, as highlighted in Ref. [241]. Analogous approaches have also been implemented in all-perovskite metasurfaces [242] and bulky WS_2_ [27] to offer direct control of light–matter interactions (Figure 4(b1–b3)). The strong coupling gives rise to polaritons in such hybrid systems as a result of the hybridization of excitons with optical BIC, which has been leveraged to enhance nonlinearity [26] and achieve high-directionality light emission [243,244]. Beyond the development of novel photonic devices, the extended lifetimes of polaritons accumulated in quasi-BIC have also advanced research in the observation of collective behavior and inherent properties of Bose–Einstein condensates [32,245].

In conclusion, the incorporation of BIC into nanostructures has enhanced the performance of integrated optoelectronic devices. By transferring 2D materials onto nanostructures as adjacent layers, the synergy between BIC and 2D materials facilitates strong light–matter interactions, effectively harnessing the optoelectronic attributes of 2D materials for dynamic optical modulation and high-performance active device fabrication. Recent research on patterning 2D materials directly into metasurfaces presents a promising avenue for further exploring exotic polaritons in 2D materials and can also be extended to other van der Waals materials, such as black phosphorus and hexagonal boron nitride [27].

Despite vibrant advancements in BIC research, several practical challenges still warrant further investigation, including the fabrication robustness of BIC metasurface. Although symmetry-protected BIC in metasurfaces has garnered widespread attention from researchers due to its design simplicity, this type of BIC demands high fabrication precision for achieving a high-quality factor, which is challenging when a defect is introduced at the subwavelength scale. When accounting for fabrication errors, particularly the random geometric variations, metasurfaces of different shapes suffer from varying degrees of degradation [246]. Therefore, for symmetry-protected BIC in metasurfaces with smaller unit structures, an in-depth discussion on error analysis or comprehensive experimental validation is essential to evaluate practical device performance. We believe that as the photonic community delves deeper into the intriguing properties of BIC, its vast potential will unfold, heralding a shift in the limits of what is possible within integrated photonics.

## Data Availability

Data sharing not applicable.

## References

[1] Azzam S.I., Kildishev A.V. (2021). Photonic bound states in the continuum: From basics to applications. Adv. Opt. Mater..

[2] Hsu C.W., Zhen B., Stone A.D., Joannopoulos J.D., Soljačić M. (2016). Bound states in the continuum. Nat. Rev. Mater..

[3] Joseph S., Pandey S., Sarkar S., Joseph J. (2021). Bound states in the continuum in resonant nanostructures: An overview of engineered materials for tailored applications. Nanophotonics.

[4] von Neumann J., Wigner E.P. (1993). Über merkwürdige diskrete Eigenwerte. The Collected Works of Eugene Paul Wigner: Part A: The Scientific Papers.

[5] Stillinger F.H., Herrick D.R. (1975). Bound states in the continuum. Phys. Rev. A.

[6] Cumpsty N.A., Whitehead D. (1971). The excitation of acoustic resonances by vortex shedding. J. Sound Vib..

[7] Parker R. (1966). Resonance effects in wake shedding from parallel plates: Some experimental observations. J. Sound Vib..

[8] Parker R. (1967). Resonance effects in wake shedding from parallel plates: Calculation of resonant frequencies. J. Sound Vib..

[9] Huang L., Jia B., Pilipchuk A.S., Chiang Y., Huang S., Li J., Shen C., Bulgakov E.N., Deng F., Powell D.A. (2022). General Framework of Bound States in the Continuum in an Open Acoustic Resonator. Phys. Rev. Appl..

[10] Cobelli P., Pagneux V., Maurel A., Petitjeans P. (2011). Experimental study on water-wave trapped modes. J. Fluid Mech..

[11] Craster R.V., Kaplunov J. (2013). Dynamic Localization Phenomena in Elasticity, Acoustics and Electromagnetism.

[12] Cobelli P., Pagneux V., Maurel A., Petitjeans P. (2009). Experimental observation of trapped modes in a water wave channel. Europhys. Lett..

[13] Marinica D., Borisov A., Shabanov S. (2008). Bound states in the continuum in photonics. Phys. Rev. Lett..

[14] Plotnik Y., Peleg O., Dreisow F., Heinrich M., Nolte S., Szameit A., Segev M. (2011). Experimental observation of optical bound states in the continuum. Phys. Rev. Lett..

[15] Gao X., Zhen B., Soljacic M., Chen H., Hsu C.W. (2019). Bound states in the continuum in fiber Bragg gratings. ACS Photonics.

[16] Bulgakov E.N., Sadreev A.F. (2008). Bound states in the continuum in photonic waveguides inspired by defects. Phys. Rev. B.

[17] Hsu C.W., Zhen B., Lee J., Chua S.-L., Johnson S.G., Joannopoulos J.D., Soljačić M. (2013). Observation of trapped light within the radiation continuum. Nature.

[18] Zou C.L., Cui J.M., Sun F.W., Xiong X., Zou X.B., Han Z.F., Guo G.C. (2015). Guiding light through optical bound states in the continuum for ultrahigh-Q microresonators. Laser Photonics Rev..

[19] Sadrieva Z.F., Sinev I.S., Koshelev K.L., Samusev A., Iorsh I.V., Takayama O., Malureanu R., Bogdanov A.A., Lavrinenko A.V. (2017). Transition from optical bound states in the continuum to leaky resonances: Role of substrate and roughness. Acs Photonics.

[20] Jin J., Yin X., Ni L., Soljačić M., Zhen B., Peng C. (2019). Topologically enabled ultrahigh-Q guided resonances robust to out-of-plane scattering. Nature.

[21] Kodigala A., Lepetit T., Gu Q., Bahari B., Fainman Y., Kanté B. (2017). Lasing action from photonic bound states in continuum. Nature.

[22] Mohamed S., Wang J., Rekola H., Heikkinen J., Asamoah B., Shi L., Hakala T.K. (2022). Controlling topology and polarization state of lasing photonic bound states in continuum. Laser Photonics Rev..

[23] Koshelev K., Kruk S., Melik-Gaykazyan E., Choi J.-H., Bogdanov A., Park H.-G., Kivshar Y. (2020). Subwavelength dielectric resonators for nonlinear nanophotonics. Science.

[24] Wu M., Ha S.T., Shendre S., Durmusoglu E.G., Koh W.-K., Abujetas D.R., Sánchez-Gil J.A., Paniagua-Domínguez R., Demir H.V., Kuznetsov A.I. (2020). Room-temperature lasing in colloidal nanoplatelets via Mie-resonant bound states in the continuum. Nano Lett..

[25] Minkov M., Gerace D., Fan S. (2019). Doubly resonant χ (2) nonlinear photonic crystal cavity based on a bound state in the continuum. Optica.

[26] Kravtsov V., Khestanova E., Benimetskiy F.A., Ivanova T., Samusev A.K., Sinev I.S., Pidgayko D., Mozharov A.M., Mukhin I.S., Lozhkin M.S. (2020). Nonlinear polaritons in a monolayer semiconductor coupled to optical bound states in the continuum. Light Sci. Appl..

[27] Weber T., Kühner L., Sortino L., Ben Mhenni A., Wilson N.P., Kühne J., Finley J.J., Maier S.A., Tittl A. (2023). Intrinsic strong light-matter coupling with self-hybridized bound states in the continuum in van der Waals metasurfaces. Nat. Mater..

[28] Maggiolini E., Polimeno L., Todisco F., Di Renzo A., Han B., De Giorgi M., Ardizzone V., Schneider C., Mastria R., Cannavale A. (2023). Strongly enhanced light–matter coupling of monolayer WS2 from a bound state in the continuum. Nat. Mater..

[29] Romano S., Mangini M., Penzo E., Cabrini S., De Luca A.C., Rendina I., Mocella V., Zito G. (2020). Ultrasensitive surface refractive index imaging based on quasi-bound states in the continuum. ACS Nano.

[30] Huang C., Zhang C., Xiao S., Wang Y., Fan Y., Liu Y., Zhang N., Qu G., Ji H., Han J. (2020). Ultrafast control of vortex microlasers. Science.

[31] Zhang X., Liu Y., Han J., Kivshar Y., Song Q. (2022). Chiral emission from resonant metasurfaces. Science.

[32] Ardizzone V., Riminucci F., Zanotti S., Gianfrate A., Efthymiou-Tsironi M., Suàrez-Forero D., Todisco F., De Giorgi M., Trypogeorgos D., Gigli G. (2022). Polariton Bose–Einstein condensate from a bound state in the continuum. Nature.

[33] Kivshar Y. (2022). Resonant tunneling and bound states in the continuum. Low Temp. Phys..

[34] Wang T., Zhang S. (2018). Large enhancement of second harmonic generation from transition-metal dichalcogenide monolayer on grating near bound states in the continuum. Opt. Express.

[35] Zhu X., Fu J., Ding F., Jin Y., Wu A. (2018). Angle-insensitive narrowband optical absorption based on high-Q localized resonance. Sci. Rep..

[36] Vaidya S., Benalcazar W.A., Cerjan A., Rechtsman M.C. (2021). Point-defect-localized bound states in the continuum in photonic crystals and structured fibers. Phys. Rev. Lett..

[37] Zhou C., Huang L., Jin R., Xu L., Li G., Rahmani M., Chen X., Lu W., Miroshnichenko A.E. (2023). Bound states in the continuum in asymmetric dielectric metasurfaces. Laser Photonics Rev..

[38] Chen Y., Deng H., Sha X., Chen W., Wang R., Chen Y.-H., Wu D., Chu J., Kivshar Y.S., Xiao S. (2023). Observation of intrinsic chiral bound states in the continuum. Nature.

[39] Koshelev K., Bogdanov A., Kivshar Y. (2019). Meta-optics and bound states in the continuum. Sci. Bull..

[40] Tian J., Adamo G., Liu H., Wu M., Klein M., Deng J., Ang N.S.S., Paniagua-Domínguez R., Liu H., Kuznetsov A.I. (2023). Phase-Change Perovskite Microlaser with Tunable Polarization Vortex. Adv. Mater..

[41] Liu X.-J., Yu Y., Liu D., Cui Q.-L., Qi X., Chen Y., Qu G., Song L., Guo G.-P., Guo G.-C. (2023). Coupling of Photon Emitters in Monolayer WS2 with a Photonic Waveguide Based on Bound States in the Continuum. Nano Lett..

[42] Koshelev K., Favraud G., Bogdanov A., Kivshar Y., Fratalocchi A. (2019). Nonradiating photonics with resonant dielectric nanostructures. Nanophotonics.

[43] Robnik M. (1986). A simple separable Hamiltonian having bound states in the continuum. J. Phys. A Math. Gen..

[44] Corrielli G., Della Valle G., Crespi A., Osellame R., Longhi S. (2013). Observation of surface states with algebraic localization. Phys. Rev. Lett..

[45] Yao H.-Y., Wang Y.-C., HadavandMirzaee F., Chang T.-H., Her T.-H. (2023). Mechanism and tuning sensitivity of symmetry-protected resonances in high-contrast gratings. Opt. Express.

[46] Murai S., Abujetas D.R., Liu L., Castellanos G.W., Giannini V., Sánchez-Gil J.A., Tanaka K., Gómez Rivas J. (2022). Engineering bound states in the continuum at telecom wavelengths with non-bravais lattices. Laser Photonics Rev..

[47] Doiron C.F., Brener I., Cerjan A. (2022). Realizing symmetry-guaranteed pairs of bound states in the continuum in metasurfaces. Nat. Commun..

[48] Wang X., Wang J., Zhao X., Shi L., Zi J. (2022). Realizing tunable evolution of bound states in the continuum and circularly polarized points by symmetry breaking. ACS Photonics.

[49] Koshelev K., Lepeshov S., Liu M., Bogdanov A., Kivshar Y. (2018). Asymmetric metasurfaces with high-Q resonances governed by bound states in the continuum. Phys. Rev. Lett..

[50] Kupriianov A.S., Xu Y., Sayanskiy A., Dmitriev V., Kivshar Y.S., Tuz V.R. (2019). Metasurface engineering through bound states in the continuum. Phys. Rev. Appl..

[51] Cai Y., Huang Y., Zhu K., Wu H. (2021). Symmetric metasurface with dual band polarization-independent high-Q resonances governed by symmetry-protected BIC. Opt. Lett..

[52] Sakoda K. (1995). Symmetry, degeneracy, and uncoupled modes in two-dimensional photonic lattices. Phys. Rev. B.

[53] Jiang H., Han Z. (2022). Spectral stability of bound state in the continuum resonances due to thermal effect and the application as efficient thermo-optic modulators. Opt. Commun..

[54] Limonov M.F., Rybin M.V., Poddubny A.N., Kivshar Y.S. (2017). Fano resonances in photonics. Nat. Photonics.

[55] Tan T.C., Plum E., Singh R. (2020). Lattice-enhanced Fano resonances from bound states in the continuum metasurfaces. Adv. Opt. Mater..

[56] Blanchard C., Hugonin J.-P., Sauvan C. (2016). Fano resonances in photonic crystal slabs near optical bound states in the continuum. Phys. Rev. B.

[57] Li S., Zhou C., Liu T., Xiao S. (2019). Symmetry-protected bound states in the continuum supported by all-dielectric metasurfaces. Phys. Rev. A.

[58] Wang X., Li S., Zhou C. (2020). Polarization-independent toroidal dipole resonances driven by symmetry-protected BIC in ultraviolet region. Opt. Express.

[59] Romano S., Zito G., Torino S., Calafiore G., Penzo E., Coppola G., Cabrini S., Rendina I., Mocella V. (2018). Label-free sensing of ultralow-weight molecules with all-dielectric metasurfaces supporting bound states in the continuum. Photonics Res..

[60] Maksimov D.N., Gerasimov V.S., Romano S., Polyutov S.P. (2020). Refractive index sensing with optical bound states in the continuum. Opt. Express.

[61] Wang Y., Han Z., Du Y., Qin J. (2021). Ultrasensitive terahertz sensing with high-Q toroidal dipole resonance governed by bound states in the continuum in all-dielectric metasurface. Nanophotonics.

[62] Mao W., Li Y., Jiang X., Liu Z., Yang L. (2023). A whispering-gallery scanning microprobe for Raman spectroscopy and imaging. Light Sci. Appl..

[63] van Hoof N.J., Abujetas D.R., Ter Huurne S.E., Verdelli F., Timmermans G.C., Sánchez-Gil J.A., Rivas J.G. (2021). Unveiling the symmetry protection of bound states in the continuum with terahertz near-field imaging. ACS Photonics.

[64] Tittl A., Leitis A., Liu M., Yesilkoy F., Choi D.-Y., Neshev D.N., Kivshar Y.S., Altug H. (2018). Imaging-based molecular barcoding with pixelated dielectric metasurfaces. Science.

[65] Yesilkoy F., Arvelo E.R., Jahani Y., Liu M., Tittl A., Cevher V., Kivshar Y., Altug H. (2019). Ultrasensitive hyperspectral imaging and biodetection enabled by dielectric metasurfaces. Nat. Photonics.

[66] Hwang M.-S., Lee H.-C., Kim K.-H., Jeong K.-Y., Kwon S.-H., Koshelev K., Kivshar Y., Park H.-G. (2021). Ultralow-threshold laser using super-bound states in the continuum. Nat. Commun..

[67] Sidorenko M., Sergaeva O., Sadrieva Z., Roques-Carmes C., Muraev P., Maksimov D., Bogdanov A. (2021). Observation of an accidental bound state in the continuum in a chain of dielectric disks. Phys. Rev. Appl..

[68] Abujetas D.R., Olmos-Trigo J., Sánchez-Gil J.A. (2022). Tailoring Accidental Double Bound States in the Continuum in All-Dielectric Metasurfaces. Adv. Opt. Mater..

[69] Odit M., Koshelev K., Gladyshev S., Ladutenko K., Kivshar Y., Bogdanov A. (2021). Observation of supercavity modes in subwavelength dielectric resonators. Adv. Mater..

[70] Melik-Gaykazyan E., Koshelev K., Choi J.-H., Kruk S.S., Bogdanov A., Park H.-G., Kivshar Y. (2021). From Fano to quasi-BIC resonances in individual dielectric nanoantennas. Nano Lett..

[71] Zhao X., Chen C., Kaj K., Hammock I., Huang Y., Averitt R.D., Zhang X. (2020). Terahertz investigation of bound states in the continuum of metallic metasurfaces. Optica.

[72] Ovcharenko A.I., Blanchard C., Hugonin J.-P., Sauvan C. (2020). Bound states in the continuum in symmetric and asymmetric photonic crystal slabs. Phys. Rev. B.

[73] Li X., Maqbool E., Han Z. (2023). Narrowband mid-infrared thermal emitters based on the Fabry-Perot type of bound states in the continuum. Opt. Express.

[74] Friedrich H., Wintgen D. (1985). Interfering resonances and bound states in the continuum. Phys. Rev. A.

[75] Suh W., Wang Z., Fan S. (2004). Temporal coupled-mode theory and the presence of non-orthogonal modes in lossless multimode cavities. IEEE J. Quantum Electron..

[76] Fan S., Suh W., Joannopoulos J.D. (2003). Temporal coupled-mode theory for the Fano resonance in optical resonators. JOSA A.

[77] Niu J., Zhai Y., Han Q., Liu J., Yang B. (2021). Resonance-trapped bound states in the continuum in metallic THz metasurfaces. Opt. Lett..

[78] Sadrieva Z., Frizyuk K., Petrov M., Kivshar Y., Bogdanov A. (2019). Multipolar origin of bound states in the continuum. Phys. Rev. B.

[79] Zhen B., Hsu C.W., Lu L., Stone A.D., Soljačić M. (2014). Topological nature of optical bound states in the continuum. Phys. Rev. Lett..

[80] Jackson J.D. (1999). Classical Electrodynamics.

[81] Liu T., Xu R., Yu P., Wang Z., Takahara J. (2020). Multipole and multimode engineering in Mie resonance-based metastructures. Nanophotonics.

[82] Barati Sedeh H., Litchinitser N.M. (2023). Singular optics empowered by engineered optical materials. Nanophotonics.

[83] Dorodnyy A., Smajic J., Leuthold J. (2023). Mie Scattering for Photonic Devices. Laser Photonics Rev..

[84] Löschenbrand D., Mecklenbrauker C. Fast antenna characterization via a sparse spherical multipole expansion. Proceedings of the IEEE 4th International Workshop on Compressed Sensing Theory and its Applications to Radar, Sonar and Remote Sensing (CoSeRa).

[85] Gonzalez-Ovejero D., Mesa F., Craeye C. (2012). Accelerated macro basis functions analysis of finite printed antenna arrays through 2D and 3D multipole expansions. IEEE Trans. Antennas Propag..

[86] Chen W., Chen Y., Liu W. (2019). Multipolar conversion induced subwavelength high-Q Kerker supermodes with unidirectional radiations. Laser Photonics Rev..

[87] Volkovskaya I., Xu L., Huang L., Smirnov A.I., Miroshnichenko A.E., Smirnova D. (2020). Multipolar second-harmonic generation from high-Q quasi-BIC states in subwavelength resonators. Nanophotonics.

[88] Shi T., Deng Z.-L., Tu Q.-A., Cao Y., Li X. (2021). Displacement-mediated bound states in the continuum in all-dielectric superlattice metasurfaces. PhotoniX.

[89] Doeleman H.M., Monticone F., den Hollander W., Alù A., Koenderink A.F. (2018). Experimental observation of a polarization vortex at an optical bound state in the continuum. Nat. Photonics.

[90] Ota Y., Takata K., Ozawa T., Amo A., Jia Z., Kante B., Notomi M., Arakawa Y., Iwamoto S. (2020). Active topological photonics. Nanophotonics.

[91] Khanikaev A.B., Shvets G. (2017). Two-dimensional topological photonics. Nat. Photonics.

[92] Lu L., Joannopoulos J.D., Soljačić M. (2014). Topological photonics. Nat. Photonics.

[93] Kartashov Y.V., Skryabin D.V. (2019). Two-dimensional topological polariton laser. Phys. Rev. Lett..

[94] Harder T.H., Sun M., Egorov O.A., Vakulchyk I., Beierlein J., Gagel P., Emmerling M., Schneider C., Peschel U., Savenko I.G. (2021). Coherent topological polariton laser. ACS Photonics.

[95] Yang Y., Yamagami Y., Yu X., Pitchappa P., Webber J., Zhang B., Fujita M., Nagatsuma T., Singh R. (2020). Terahertz topological photonics for on-chip communication. Nat. Photonics.

[96] Bulgakov E.N., Maksimov D.N. (2017). Topological bound states in the continuum in arrays of dielectric spheres. Phys. Rev. Lett..

[97] Wang B., Liu W., Zhao M., Wang J., Zhang Y., Chen A., Guan F., Liu X., Shi L., Zi J. (2020). Generating optical vortex beams by momentum-space polarization vortices centred at bound states in the continuum. Nat. Photonics.

[98] Kang L., Wu Y., Ma X., Lan S., Werner D.H. (2022). High-harmonic optical vortex generation from photonic bound states in the continuum. Adv. Opt. Mater..

[99] Meng Y., Liu Z., Xie Z., Wang R., Qi T., Hu F., Kim H., Xiao Q., Fu X., Wu Q. (2020). Versatile on-chip light coupling and (de)multiplexing from arbitrary polarizations to controlled waveguide modes using an integrated dielectric metasurface. Photon. Res..

[100] He T., Meng Y., Liu Z., Hu F., Wang R., Li D., Yan P., Liu Q., Gong M., Xiao Q. (2021). Guided mode meta-optics: Metasurface-dressed waveguides for arbitrary mode couplers and on-chip OAM emitters with a configurable topological charge. Opt. Express.

[101] Meng Y., Chen Y., Lu L., Ding Y., Cusano A., Fan J.A., Hu Q., Wang K., Xie Z., Liu Z. (2021). Optical meta-waveguides for integrated photonics and beyond. Light Sci. Appl..

[102] Cheben P., Halir R., Schmid J.H., Atwater H.A., Smith D.R. (2018). Subwavelength integrated photonics. Nature.

[103] Yadav G., Sahu S., Kumar R., Jha R. (2022). Bound states in the continuum empower subwavelength gratings for refractometers in visible. Photonics.

[104] Li Z., Zhang X., Zhai Z., Cai Y., Ao X. (2022). Quasibound states in a one-dimensional grating for electro-optic modulation. Phys. Rev. B.

[105] Głowadzka W., Wasiak M., Czyszanowski T. (2021). True-and quasi-bound states in the continuum in one-dimensional gratings with broken up-down mirror symmetry. Nanophotonics.

[106] Joseph S., Sarkar S., Khan S., Joseph J. (2021). Exploring the optical bound state in the continuum in a dielectric grating coupled plasmonic hybrid system. Adv. Opt. Mater..

[107] Zhang N., Lu Y.Y. (2023). Robust and non-robust bound states in the continuum in rotationally symmetric periodic waveguides. Opt. Express.

[108] Maksimov D.N., Gerasimov V.S., Bogdanov A.A., Polyutov S.P. (2022). Enhanced sensitivity of an all-dielectric refractive index sensor with an optical bound state in the continuum. Phys. Rev. A.

[109] Zhang H., Wang T., Tian J., Sun J., Li S., De Leon I., Zaccaria R.P., Peng L., Gao F., Lin X. (2021). Quasi-BIC laser enabled by high-contrast grating resonator for gas detection. Nanophotonics.

[110] Barrow M., Phillips J. (2020). Polarization-independent narrowband transmittance filters via symmetry-protected modes in high contrast gratings. Opt. Lett..

[111] Huang Z.-T., Chang C.-Y., Chen K.-P., Lu T.-C. (2022). Tunable lasing direction in one-dimensional suspended high-contrast grating using bound states in the continuum. Adv. Photonics.

[112] Wang Y., Fan Y., Zhang X., Tang H., Song Q., Han J., Xiao S. (2021). Highly controllable etchless perovskite microlasers based on bound states in the continuum. ACS Nano.

[113] Liu M., Xia S., Wan W., Qin J., Li H., Zhao C., Bi L., Qiu C.-W. (2023). Broadband mid-infrared non-reciprocal absorption using magnetized gradient epsilon-near-zero thin films. Nat. Mater..

[114] Ning T., Li X., Zhao Y., Yin L., Huo Y., Zhao L., Yue Q. (2020). Giant enhancement of harmonic generation in all-dielectric resonant waveguide gratings of quasi-bound states in the continuum. Opt. Express.

[115] Maslova E.E., Rybin M.V., Bogdanov A.A., Sadrieva Z.F. (2021). Bound states in the continuum in periodic structures with structural disorder. Nanophotonics.

[116] Liu X., Zhang C., Hu J., Han H. (2023). Dual-band refractive index sensor with cascaded asymmetric resonant compound grating based on bound states in the continuum. Opt. Express.

[117] Liu C., Bai Y., Zhou J., Chen J., Qiao L. (2021). Refractive index sensing by asymmetric dielectric gratings with both bound states in the continuum and guided mode resonances. Opt. Express.

[118] Hemmati H., Magnusson R. (2019). Resonant dual-grating metamembranes supporting spectrally narrow bound states in the continuum. Adv. Opt. Mater..

[119] Liu Z., Meng Y., Zhou Z., Wang L., He T., Li D., Yan P., Gong M., Xiao Q. (2022). Broadband nanostructured fiber mode convertors enabled by inverse design. Opt. Express.

[120] Isaacs S., Hajoj A., Abutoama M., Kozlovsky A., Golan E., Abdulhalim I. (2019). Resonant grating without a planar waveguide layer as a refractive index sensor. Sensors.

[121] Abdulhalim I. (2009). Optimized guided mode resonant structure as thermooptic sensor and liquid crystal tunable filter. Chin. Opt. Lett..

[122] Sang T., Cai S., Wang Z. (2011). Guided-mode resonance filter with an antireflective surface consisting of a buffer layer with refractive index equal to that of the grating. J. Mod. Opt..

[123] Sahoo P.K., Sarkar S., Joseph J. (2017). High sensitivity guided-mode-resonance optical sensor employing phase detection. Sci. Rep..

[124] Hu F., Jia W., Meng Y., Gong M., Yang Y. (2019). High-contrast optical switching using an epsilon-near-zero material coupled to a Bragg microcavity. Opt. Express.

[125] Wu F., Wu J., Guo Z., Jiang H., Sun Y., Li Y., Ren J., Chen H. (2019). Giant enhancement of the Goos-Hänchen shift assisted by quasibound states in the continuum. Phys. Rev. Appl..

[126] Wu F., Luo M., Wu J., Fan C., Qi X., Jian Y., Liu D., Xiao S., Chen G., Jiang H. (2021). Dual quasibound states in the continuum in compound grating waveguide structures for large positive and negative Goos-Hänchen shifts with perfect reflection. Phys. Rev. A.

[127] Wu F., Liu T., Long Y., Xiao S., Chen G. (2023). Giant photonic spin Hall effect empowered by polarization-dependent quasibound states in the continuum in compound grating waveguide structures. Phys. Rev. B.

[128] Kim K.S., Lee D., Chang C.S., Seo S., Hu Y., Kim H., Shin J., Lee J.-H., Lee S., Kim J.S. (2023). Non-epitaxial single-crystal 2D material growth by geometrical confinement. Nature.

[129] Bae S.-H., Kum H., Kong W., Kim Y., Choi C., Lee B., Lin P., Park Y., Kim J. (2019). Integration of bulk materials with two-dimensional materials for physical coupling and applications. Nat. Mater..

[130] Kim H., Lee S., Shin J., Zhu M., Akl M., Lu K., Han N.M., Baek Y., Chang C.S., Suh J.M. (2022). Graphene nanopattern as a universal epitaxy platform for single-crystal membrane production and defect reduction. Nat. Nanotechnol..

[131] Kong W., Kum H., Bae S.-H., Shim J., Kim H., Kong L., Meng Y., Wang K., Kim C., Kim J. (2019). Path towards graphene commercialization from lab to market. Nat. Nanotechnol..

[132] Meng Y., Feng J., Han S., Xu Z., Mao W., Zhang T., Kim J., Roh I., Zhao Y., Kim D.-H. (2023). Photonic van der Waals integration from 2D materials to 3D nanomembranes. Nat. Rev. Mater..

[133] Zhang Q., Hu G., Ma W., Li P., Krasnok A., Hillenbrand R., Alù A., Qiu C.-W. (2021). Interface nano-optics with van der Waals polaritons. Nature.

[134] Liu Z., Meng Y., Hu F., Xiao Q., Yan P., Gong M. (2019). Largely Tunable Terahertz Circular Polarization Splitters Based on Patterned Graphene Nanoantenna Arrays. IEEE Photonics J..

[135] Meng Y., Zhong H., Xu Z., He T., Kim J.S., Han S., Kim S., Park S., Shen Y., Gong M. (2023). Functionalizing nanophotonic structures with 2D van der Waals materials. Nanoscale Horiz..

[136] Xia F., Wang H., Xiao D., Dubey M., Ramasubramaniam A. (2014). Two-dimensional material nanophotonics. Nat. Photonics.

[137] Wu F., Liu D., Xiao S. (2021). Bandwidth-tunable near-infrared perfect absorption of graphene in a compound grating waveguide structure supporting quasi-bound states in the continuum. Opt. Express.

[138] Meng Y., Hu F., Shen Y., Yang Y., Xiao Q., Fu X., Gong M. (2018). Ultracompact graphene-assisted tunable waveguide couplers with high directivity and mode selectivity. Sci. Rep..

[139] SoljaČiĆ M., Joannopoulos J.D. (2004). Enhancement of nonlinear effects using photonic crystals. Nat. Mater..

[140] Noda S., Fujita M., Asano T. (2007). Spontaneous-emission control by photonic crystals and nanocavities. Nat. Photonics.

[141] Joannopoulos J.D., Johnson S.G., Winn J.N., Meade R.D. (2008). Molding the Flow of Light.

[142] Zhang Z., Yang J., Du T., Ma H., Jiang X. (2022). Tailoring bound states in the continuum in symmetric photonic crystal slabs by coupling strengths. Opt. Express.

[143] Lee S.-G., Kim S.-H., Kee C.-S. (2020). Bound states in the continuum (BIC) accompanied by avoided crossings in leaky-mode photonic lattices. Nanophotonics.

[144] Dai S., Hu P., Han D. (2020). Near-field analysis of bound states in the continuum in photonic crystal slabs. Opt. Express.

[145] Liu Z., Li X., Chen C., Wang X., Gao W., Ye W., Li L., Liu J. (2023). Bound states in the continuum in asymmetric one-dimensional photonic crystal systems guided by anisotropy. Opt. Express.

[146] Wang W., Srivastava Y.K., Tan T.C., Wang Z., Singh R. (2023). Brillouin zone folding driven bound states in the continuum. Nat. Commun..

[147] Kang M., Zhang S., Xiao M., Xu H. (2021). Merging bound states in the continuum at off-high symmetry points. Phys. Rev. Lett..

[148] Kang M., Mao L., Zhang S., Xiao M., Xu H., Chan C.T. (2022). Merging bound states in the continuum by harnessing higher-order topological charges. Light Sci. Appl..

[149] Wan S., Wang K., Wang F., Guan C., Li W., Liu J., Bogdanov A., Belov P.A., Shi J. (2022). Topologically enabled ultrahigh-Q chiroptical resonances by merging bound states in the continuum. Opt. Lett..

[150] Kang M., Zhang Z., Wu T., Zhang X., Xu Q., Krasnok A., Han J., Alù A. (2022). Coherent full polarization control based on bound states in the continuum. Nat. Commun..

[151] Qin H., Su Z., Liu M., Zeng Y., Tang M.-C., Li M., Shi Y., Huang W., Qiu C.-W., Song Q. (2023). Arbitrarily polarized bound states in the continuum with twisted photonic crystal slabs. Light Sci. Appl..

[152] Zong X., Li L., Liu Y. (2022). Bound states in the continuum in all-van der Waals photonic crystals: A route enabling electromagnetically induced transparency. Opt. Express.

[153] Fan Y., Shen N.-H., Zhang F., Zhao Q., Wu H., Fu Q., Wei Z., Li H., Soukoulis C.M. (2019). Graphene Plasmonics: A Platform for 2D Optics. Adv. Opt. Mater..

[154] Miao J., Xu Z., Li Q., Bowman A., Zhang S., Hu W., Zhou Z., Wang C. (2017). Vertically Stacked and Self-Encapsulated van der Waals Heterojunction Diodes Using Two-Dimensional Layered Semiconductors. ACS Nano.

[155] Vertchenko L., DeVault C., Malureanu R., Mazur E., Lavrinenko A. (2021). Near-zero index photonic crystals with directive bound states in the continuum. Laser Photonics Rev..

[156] Hu F., Li L., Liu Y., Meng Y., Gong M., Yang Y. (2021). Two-plasmon spontaneous emission from a nonlocal epsilon-near-zero material. Commun. Phys..

[157] Marpaung D., Yao J., Capmany J. (2019). Integrated microwave photonics. Nat. Photonics.

[158] Wang C., Fu Z., Mao W., Qie J., Stone A.D., Yang L. (2023). Non-Hermitian optics and photonics: From classical to quantum. Adv. Opt. Photonics.

[159] Faraon A., Barclay P.E., Santori C., Fu K.-M.C., Beausoleil R.G. (2011). Resonant enhancement of the zero-phonon emission from a colour centre in a diamond cavity. Nat. Photonics.

[160] Hausmann B.J., Shields B., Quan Q., Maletinsky P., McCutcheon M., Choy J.T., Babinec T.M., Kubanek A., Yacoby A., Lukin M.D. (2012). Integrated diamond networks for quantum nanophotonics. Nano Lett..

[161] Gomis-Bresco J., Artigas D., Torner L. (2017). Anisotropy-induced photonic bound states in the continuum. Nat. Photonics.

[162] Gu Z., Jiang S., Liu C., Zhang N. (2023). Robust bound states in the continuum in a dual waveguide system. Photonics Res..

[163] Huang H., Overvig A.C., Xu Y., Malek S.C., Tsai C.-C., Alù A., Yu N. (2023). Leaky-wave metasurfaces for integrated photonics. Nat. Nanotechnol..

[164] Overvig A., Yu N., Alù A. (2021). Chiral Quasi-Bound States in the Continuum. Phys. Rev. Lett..

[165] Feng Z., Sun X. (2023). Experimental Observation of Dissipatively Coupled Bound States in the Continuum on an Integrated Photonic Platform. Laser Photonics Rev..

[166] Yu Z., Xi X., Ma J., Tsang H.K., Zou C.-L., Sun X. (2019). Photonic integrated circuits with bound states in the continuum. Optica.

[167] Yu Z., Wang Y., Sun B., Tong Y., Xu J.B., Tsang H.K., Sun X. (2019). Hybrid 2D-Material Photonics with Bound States in the Continuum. Adv. Opt. Mater..

[168] Yu Z., Sun X. (2020). Acousto-optic modulation of photonic bound state in the continuum. Light Sci. Appl..

[169] Ye F., Yu Y., Xi X., Sun X. (2022). Second-Harmonic Generation in Etchless Lithium Niobate Nanophotonic Waveguides with Bound States in the Continuum. Laser Photonics Rev..

[170] Yu Z., Tong Y., Tsang H.K., Sun X. (2020). High-dimensional communication on etchless lithium niobate platform with photonic bound states in the continuum. Nat. Commun..

[171] Jankowski M., Langrock C., Desiatov B., Marandi A., Wang C., Zhang M., Phillips C.R., Lončar M., Fejer M. (2020). Ultrabroadband nonlinear optics in nanophotonic periodically poled lithium niobate waveguides. Optica.

[172] Hu G., Hong X., Wang K., Wu J., Xu H.-X., Zhao W., Liu W., Zhang S., Garcia-Vidal F., Wang B. (2019). Coherent steering of nonlinear chiral valley photons with a synthetic Au–WS2 metasurface. Nat. Photonics.

[173] Boes A., Corcoran B., Chang L., Bowers J., Mitchell A. (2018). Status and potential of lithium niobate on insulator (LNOI) for photonic integrated circuits. Laser Photonics Rev..

[174] Li X., Ma J., Liu S., Huang P., Chen B., Wei D., Liu J. (2022). Efficient second harmonic generation by harnessing bound states in the continuum in semi-nonlinear etchless lithium niobate waveguides. Light Sci. Appl..

[175] Meng Y., Ye S., Shen Y., Xiao Q., Fu X., Lu R., Liu Y., Gong M. (2018). Waveguide engineering of graphene optoelectronics—Modulators and polarizers. IEEE Photonics J..

[176] Meng Y., Lu R., Shen Y., Liu Y., Gong M. (2017). Ultracompact graphene-assisted ring resonator optical router. Opt. Commun..

[177] Liu C.-h., Zheng J., Chen Y., Fryett T., Majumdar A. (2019). Van der Waals materials integrated nanophotonic devices [Invited]. Opt. Mater. Express.

[178] Yu Y., Sakanas A., Zali A.R., Semenova E., Yvind K., Mørk J. (2021). Ultra-coherent Fano laser based on a bound state in the continuum. Nat. Photonics.

[179] Nguyen T.G., Ren G., Schoenhardt S., Knoerzer M., Boes A., Mitchell A. (2019). Ridge resonance in silicon photonics harnessing bound states in the continuum. Laser Photonics Rev..

[180] Lei F., Ye Z., Twayana K., Gao Y., Girardi M., Helgason Ó.B., Zhao P. (2023). Hyperparametric oscillation via bound states in the continuum. Phys. Rev. Lett..

[181] Xu H., Shi Y. (2020). Diffraction engineering for silicon waveguide grating antenna by harnessing bound state in the continuum. Nanophotonics.

[182] Wang S., Wen S., Deng Z.-L., Li X., Yang Y. (2023). Metasurface-Based Solid Poincar\’e Sphere Polarizer. Phys. Rev. Lett..

[183] Meng Y., Hu F., Liu Z., Xie P., Shen Y., Xiao Q., Fu X., Bae S.-H., Gong M. (2019). Chip-integrated metasurface for versatile and multi-wavelength control of light couplings with independent phase and arbitrary polarization. Opt. Express.

[184] Guo Y., Pu M., Li X., Ma X., Song S., Zhao Z., Luo X. (2018). Chip-Integrated Geometric Metasurface As a Novel Platform for Directional Coupling and Polarization Sorting by Spin–Orbit Interaction. IEEE J. Sel. Top. Quantum Electron..

[185] Lin L., Hu J., Dagli S., Dionne J.A., Lawrence M. (2023). Universal Narrowband Wavefront Shaping with High Quality Factor Meta-Reflect-Arrays. Nano Lett..

[186] Kim G., Kim Y., Yun J., Moon S.-W., Kim S., Kim J., Park J., Badloe T., Kim I., Rho J. (2022). Metasurface-driven full-space structured light for three-dimensional imaging. Nat. Commun..

[187] Liu C., Ma Q., Luo Z.J., Hong Q.R., Xiao Q., Zhang H.C., Miao L., Yu W.M., Cheng Q., Li L. (2022). A programmable diffractive deep neural network based on a digital-coding metasurface array. Nat. Electron..

[188] Jung C., Kim G., Jeong M., Jang J., Dong Z., Badloe T., Yang J.K., Rho J. (2021). Metasurface-driven optically variable devices. Chem. Rev..

[189] Li J., Li J., Zheng C., Yue Z., Wang S., Li M., Zhao H., Zhang Y., Yao J. (2021). Free switch between bound states in the continuum (BIC) and quasi-BIC supported by graphene-metal terahertz metasurfaces. Carbon.

[190] Luo M., Zhou Y., Zhao X., Li Y., Guo Z., Yang X., Zhang M., Wang Y., Wu X. (2022). Label-Free Bound-States-in-the-Continuum Biosensors. Biosensors.

[191] Srivastava Y.K., Ako R.T., Gupta M., Bhaskaran M., Sriram S., Singh R. (2019). Terahertz sensing of 7 nm dielectric film with bound states in the continuum metasurfaces. Appl. Phys. Lett..

[192] Chen X., Fan W., Yan H. (2020). Toroidal dipole bound states in the continuum metasurfaces for terahertz nanofilm sensing. Opt. Express.

[193] Cen W., Lang T., Hong Z., Liu J., Xiao M., Zhang J., Yu Z. (2022). Ultrasensitive flexible terahertz plasmonic metasurface sensor based on bound states in the continuum. IEEE Sens. J..

[194] Tan T.C., Srivastava Y.K., Ako R.T., Wang W., Bhaskaran M., Sriram S., Al-Naib I., Plum E., Singh R. (2021). Active control of nanodielectric-induced THz quasi-BIC in flexible metasurfaces: A platform for modulation and sensing. Adv. Mater..

[195] Wang J., Kühne J., Karamanos T., Rockstuhl C., Maier S.A., Tittl A. (2021). All-dielectric crescent metasurface sensor driven by bound states in the continuum. Adv. Funct. Mater..

[196] Liu Z., Wang L., Meng Y., He T., He S., Yang Y., Wang L., Tian J., Li D., Yan P. (2022). All-fiber high-speed image detection enabled by deep learning. Nat. Commun..

[197] Wang L., Yang Y., Liu Z., Tian J., Meng Y., Qi T., He T., Li D., Yan P., Gong M. (2022). High-Speed All-Fiber Micro-Imaging with Large Depth of Field. Laser Photonics Rev..

[198] Wang L., Qi T., Liu Z., Meng Y., Li D., Yan P., Gong M., Xiao Q. (2022). Complex pattern transmission through multimode fiber under diverse light sources. APL Photonics.

[199] Jahani Y., Arvelo E.R., Yesilkoy F., Koshelev K., Cianciaruso C., De Palma M., Kivshar Y., Altug H. (2021). Imaging-based spectrometer-less optofluidic biosensors based on dielectric metasurfaces for detecting extracellular vesicles. Nat. Commun..

[200] Wang R., Xu L., Huang L., Zhang X., Ruan H., Yang X., Lou J., Chang C., Du X. (2023). Ultrasensitive Terahertz Biodetection Enabled by Quasi-BIC-Based Metasensors. Small.

[201] Wang R., Xu L., Wang J., Sun L., Jiao Y., Meng Y., Chen S., Chang C., Fan C. (2021). Electric Fano resonance-based terahertz metasensors. Nanoscale.

[202] Hu Y., Xie S., Bai C., Shen W., Yang J. (2022). Quasi-Bound States in the Continuum Enabled Strong Terahertz Chiroptical Response in Bilayer Metallic Metasurfaces. Crystals.

[203] Zhou C., Qu X., Xiao S., Fan M. (2020). Imaging through a fano-resonant dielectric metasurface governed by quasi--bound states in the continuum. Phys. Rev. Appl..

[204] Hu F., Chen S., Wang R., Meng Y., Liu Q., Gong M. (2021). Tunable extreme energy transfer of terahertz waves with graphene in a nested cavity. Opt. Express.

[205] Zhang L., Zhang D., Jin L., Liu B., Meng H., Tang X., Li M., Liu S., Zhong Z., Zhang H. (2021). Fabrication and broadband ferromagnetic resonance studies of freestanding polycrystalline yttrium iron garnet thin films. APL Mater..

[206] Wang X., Wang X., Yao Z., Guo G., Jia Y., He Y., Jin R., Lang Y., You J., Ren Q. (2023). Digital imaging through terahertz multifrequency programmable metasurface based on BIC. Opt. Mater..

[207] Koshelev K., Tang Y., Li K., Choi D.-Y., Li G., Kivshar Y. (2019). Nonlinear metasurfaces governed by bound states in the continuum. Acs Photonics.

[208] Xu L., Zangeneh Kamali K., Huang L., Rahmani M., Smirnov A., Camacho-Morales R., Ma Y., Zhang G., Woolley M., Neshev D. (2019). Dynamic nonlinear image tuning through magnetic dipole quasi-BIC ultrathin resonators. Adv. Sci..

[209] Fang C., Yang Q., Yuan Q., Gu L., Gan X., Shao Y., Liu Y., Han G., Hao Y. (2022). Efficient second-harmonic generation from silicon slotted nanocubes with bound states in the continuum. Laser Photonics Rev..

[210] Zhang X., He L., Gan X., Huang X., Du Y., Zhai Z., Li Z., Zheng Y., Chen X., Cai Y. (2022). Quasi-Bound States in the Continuum Enhanced Second-Harmonic Generation in Thin-Film Lithium Niobate. Laser Photonics Rev..

[211] Zograf G., Koshelev K., Zalogina A., Korolev V., Hollinger R., Choi D.-Y., Zuerch M., Spielmann C., Luther-Davies B., Kartashov D. (2022). High-harmonic generation from resonant dielectric metasurfaces empowered by bound states in the continuum. Acs Photonics.

[212] Anthur A.P., Zhang H., Paniagua-Dominguez R., Kalashnikov D.A., Ha S.T., Maß T.W., Kuznetsov A.I., Krivitsky L. (2020). Continuous wave second harmonic generation enabled by quasi-bound-states in the continuum on gallium phosphide metasurfaces. Nano Lett..

[213] Santiago-Cruz T., Gennaro S.D., Mitrofanov O., Addamane S., Reno J., Brener I., Chekhova M.V. (2022). Resonant metasurfaces for generating complex quantum states. Science.

[214] He F., Liu J., Pan G., Shu F., Jing X., Hong Z. (2021). Analogue of electromagnetically induced transparency in an all-dielectric double-layer metasurface based on bound states in the continuum. Nanomaterials.

[215] Zeng T.-Y., Liu G.-D., Wang L.-L., Lin Q. (2021). Light-matter interactions enhanced by quasi-bound states in the continuum in a graphene-dielectric metasurface. Opt. Express.

[216] Chen X., Fan W. (2020). Tunable bound states in the continuum in all-dielectric terahertz metasurfaces. Nanomaterials.

[217] Li C., Cheng H., Luo X., Cheng Z., Zhai X. (2022). A High Quality-Factor Optical Modulator with Hybrid Graphene-Dielectric Metasurface Based on the Quasi-Bound States in the Continuum. Micromachines.

[218] Chen Y., Liu Z., Li Y., Hu Z., Wu J., Wang J. (2022). Adjustable converter of bound state in the continuum basing on metal-graphene hybrid metasurfaces. Opt. Express.

[219] Bernhardt N., Koshelev K., White S.J., Meng K.W.C., Froch J.E., Kim S., Tran T.T., Choi D.-Y., Kivshar Y., Solntsev A.S. (2020). Quasi-BIC resonant enhancement of second-harmonic generation in WS2 monolayers. Nano Lett..

[220] Muhammad N., Chen Y., Qiu C.-W., Wang G.P. (2021). Optical bound states in continuum in MoS2-based metasurface for directional light emission. Nano Lett..

[221] Zhou J., Lin J., Huang X., Zhou Y., Chen Y., Xia J., Wang H., Xie Y., Yu H., Lei J. (2018). A library of atomically thin metal chalcogenides. Nature.

[222] Wang Q., Plum E., Yang Q., Zhang X., Xu Q., Xu Y., Han J., Zhang W. (2018). Reflective chiral meta-holography: Multiplexing holograms for circularly polarized waves. Light Sci. Appl..

[223] Zhang Y., Oka T., Suzuki R., Ye J., Iwasa Y. (2014). Electrically switchable chiral light-emitting transistor. Science.

[224] Kim Y.-H., Zhai Y., Lu H., Pan X., Xiao C., Gaulding E.A., Harvey S.P., Berry J.J., Vardeny Z.V., Luther J.M. (2021). Chiral-induced spin selectivity enables a room-temperature spin light-emitting diode. Science.

[225] Forbes A., de Oliveira M., Dennis M.R. (2021). Structured light. Nat. Photonics.

[226] Mattioli F., Mazzeo G., Longhi G., Abbate S., Pellegrini G., Mogni E., Celebrano M., Finazzi M., Duò L., Zanchi C.G. (2020). Plasmonic superchiral lattice resonances in the mid-infrared. Acs Photonics.

[227] Tang Y., Liang Y., Yao J., Chen M.K., Lin S., Wang Z., Zhang J., Huang X.G., Yu C., Tsai D.P. (2023). Chiral bound states in the continuum in plasmonic metasurfaces. Laser Photonics Rev..

[228] Shi T., Deng Z.-L., Geng G., Zeng X., Zeng Y., Hu G., Overvig A., Li J., Qiu C.-W., Alù A. (2022). Planar chiral metasurfaces with maximal and tunable chiroptical response driven by bound states in the continuum. Nat. Commun..

[229] Gorkunov M.V., Antonov A.A., Tuz V.R., Kupriianov A.S., Kivshar Y.S. (2021). Bound states in the continuum underpin near-lossless maximum chirality in dielectric metasurfaces. Adv. Opt. Mater..

[230] Yin X., Jin J., Soljačić M., Peng C., Zhen B. (2020). Observation of topologically enabled unidirectional guided resonances. Nature.

[231] Li A., Wei H., Cotrufo M., Chen W., Mann S., Ni X., Xu B., Chen J., Wang J., Fan S. (2023). Exceptional points and non-Hermitian photonics at the nanoscale. Nat. Nanotechnol..

[232] Meudt M., Bogiadzi C., Wrobel K., Görrn P. (2020). Hybrid photonic–plasmonic bound states in continuum for enhanced light manipulation. Adv. Opt. Mater..

[233] Dubrovkin A.M., Qiang B., Krishnamoorthy H.N.S., Zheludev N.I., Wang Q.J. (2018). Ultra-confined surface phonon polaritons in molecular layers of van der Waals dielectrics. Nat. Commun..

[234] Zhou Y., Guo Z., Zhao X., Wang F., Yu Z., Chen Y., Liu Z., Zhang S., Sun S., Wu X. (2022). Dual-Quasi Bound States in the Continuum Enabled Plasmonic Metasurfaces. Adv. Opt. Mater..

[235] Xiao X., Lu Y., Jiang J., Chen Y. (2022). Manipulation of optical bound states in the continuum in a metal-dielectric hybrid nanostructure. Photonics Res..

[236] Aigner A., Tittl A., Wang J., Weber T., Kivshar Y., Maier S.A., Ren H. (2022). Plasmonic bound states in the continuum to tailor light-matter coupling. Sci. Adv..

[237] Zhou Q., Fu Y., Liu J., Yan H., Chen H., Gao L., Jiang J.H., Xu Y. (2022). Plasmonic bound states in the continuum in compact nanostructures. Adv. Opt. Mater..

[238] Lin H., Song Y., Huang Y., Kita D., Deckoff-Jones S., Wang K., Li L., Li J., Zheng H., Luo Z. (2017). Chalcogenide glass-on-graphene photonics. Nat. Photonics.

[239] Xie P., Liang Z., Jia T., Li D., Chen Y., Chang P., Zhang H., Wang W. (2021). Strong coupling between excitons in a two-dimensional atomic crystal and quasibound states in the continuum in a two-dimensional all-dielectric asymmetric metasurface. Phys. Rev. B.

[240] Koshelev K., Sychev S., Sadrieva Z.F., Bogdanov A.A., Iorsh I. (2018). Strong coupling between excitons in transition metal dichalcogenides and optical bound states in the continuum. Phys. Rev. B.

[241] Al-Ani I.A., As’ Ham K., Huang L., Miroshnichenko A.E., Hattori H.T. (2021). Enhanced strong coupling of TMDC monolayers by bound state in the continuum. Laser Photonics Rev..

[242] Al-Ani I.A., As’ Ham K., Huang L., Miroshnichenko A.E., Lei W., Hattori H.T. (2022). Strong coupling of exciton and high-Q mode in all-perovskite metasurfaces. Adv. Opt. Mater..

[243] Wang Y., Tian J., Klein M., Adamo G., Ha S.T., Soci C. (2023). Directional Emission from Electrically Injected Exciton–Polaritons in Perovskite Metasurfaces. Nano Lett..

[244] Kim S., Woo B.H., An S.-C., Lim Y., Seo I.C., Kim D.-S., Yoo S., Park Q.-H., Jun Y.C. (2021). Topological control of 2D perovskite emission in the strong coupling regime. Nano Lett..

[245] Grudinina A., Efthymiou-Tsironi M., Ardizzone V., Riminucci F., Giorgi M.D., Trypogeorgos D., Baldwin K., Pfeiffer L., Ballarini D., Sanvitto D. (2023). Collective excitations of a bound-in-the-continuum condensate. Nat. Commun..

[246] Kühne J., Wang J., Weber T., Kühner L., Maier S.A., Tittl A. (2021). Fabrication robustness in BIC metasurfaces. Nanophotonics.

